# Síndrome neuroléptico maligno asociado con intoxicación aguda por un organofosforado: reporte de caso

**DOI:** 10.7705/biomedica.6428

**Published:** 2022-09-02

**Authors:** Nancy Y. Angulo, Paula A. Castaño, Crhistian C. Gómez, Santiago Quintero

**Affiliations:** 1 Toxicología Clínica y Farmacovigilancia, IPS Universitaria Clínica León XIII, Medellín, Colombia IPS Universitaria Clínica León XIII Medellín Colombia; 2 Sección de Medicina de Urgencias, Facultad de Medicina, Universidad de Antioquia, Medellín, Colombia Universidad de Antioquia Universidad de Antioquia Medellín Colombia

**Keywords:** síndrome neuroléptico maligno, insecticidas organofosforados, colinesterasas, bromocriptina, fiebre, rabdomiólisis, Neuroleptic malignant syndrome, insecticides, organophosphate, cholinesterases, bromocriptine, fever, rhabdomyolysis

## Abstract

El síndrome neuroléptico maligno es una condición clínica rara y potencialmente letal que frecuentemente se asocia con el uso de antipsicóticos. En la literatura especializada se encontró únicamente un reporte de caso relacionado con la ingestión de organofosforados. Se presenta un paciente con un cuadro clínico correspondiente al síndrome neuroléptico maligno posterior a la ingestión de clorpirifós. Como resultado de un intento de suicidio con el mencionado organofosforado, el hombre de 57 años presentó deterioro agudo del estado de consciencia, evolución neurológica tórpida e inestabilidad autonómica asociada a rigidez e hipertermia persistentes, así como incremento de la creatina-fosfocinasa (*creatine phosphokinase*, CPK). Se le administró tratamiento con bromocriptina, con lo cual el cuadro clínico remitió, y fue dado de alta sin secuelas. El diagnóstico del síndrome neuroléptico maligno es clínico y debe contemplarse en cualquier caso de exposición a sustancias que puedan resultar en una desregulación de la neurotransmisión dopaminérgica, con el fin de iniciar el tratamiento oportuno y contrarrestar efectivamente los efectos.

## Abstract (en)

Neuroleptic malignant syndrome is a rare and potentially fatal clinical condition frequently associated with the use of antipsychotics. In the literature, there is only one case report associated with the intake of organophosphates. We present the case of a patient who presented with a clinical picture compatible with neuroleptic malignant syndrome, after the ingestion of an organophosphate (chlorpyrifos). A 57-year-old man who consulted for attempted suicide, acute deterioration of consciousness, torpid neurological evolution, and associated autonomic instability associated with rigidity, persistent hyperthermia, and elevated CPK. Bromocriptine treatment was offered, which resolved the clinical picture. The association with the ingestion of an organophosphate was established, and he was discharged without sequelae. The diagnosis of neuroleptic malignant syndrome is clinical and should be considered in any case of exposure to substances that may lead to dysregulation of dopaminergic neurotransmission in order to initiate timely therapy and impact outcomes.

La intoxicación aguda por organofosforados es un problema mundial de salud ^1^. Las manifestaciones clínicas se asocian con la unión del tóxico al residuo de serina, lo que inhibe de forma irreversible la acción de la acetilcolinesterasa por fosforilación, con la consecuente estimulación excesiva de los receptores muscarínicos y nicotínicos. Esto produce una crisis colinérgica aguda que puede cursar con complicaciones específicas, como el síndrome intermedio y la neuropatía retardada ^1^^,^^2^. En un caso reportado, dicha intoxicación se asoció con la aparición del síndrome neuroléptico maligno ^2^. Aquí se presenta el caso de un paciente de 57 años que presentó este síndrome después de una intoxicación aguda por organofosforado.

## Reporte de caso

Se trató de un paciente de 57 años con antecedentes de tabaquismo activo, originario de la zona rural de un municipio de Colombia. Tras la ingestión de un tóxico (hasta ese momento desconocido) con intención suicida en el contexto de consumo de alcohol, ingresó al hospital local durante las primeras dos horas de evolución de un cuadro agudo de confusión, bradicardia, diaforesis e hipertensión arterial, asociado con acidosis metabólica. Recibió dos ampollas de atropina para el manejo del “toxidrome” colinérgico y la bradicardia, y se le hizo lavado gástrico con carbón activado. Aproximadamente a las cuatro horas de la ingestión, presentó alteración del grado de conciencia, así como disnea, por lo que se le practicó intubación orotraqueal y fue trasladado a un centro de referencia.

Inicialmente, se desconocía el compuesto ingerido, por lo que se diagnosticó una intoxicación de origen incierto, probablemente relacionada con sedantes y alcohol. A su llegada al centro de salud, siete horas después de la ingestión, el paciente tenía presión arterial de 103/69 mm Hg, frecuencia cardiaca de 84 latidos por minuto, saturación de oxígeno del 93 % con soporte respiratorio, y un puntaje de (-) 4 puntos en la *Richmond Agitation-Sedation Scale* (RASS). Se ordenó el tratamiento con líquidos endovenosos. Los resultados de laboratorio evidenciaron un recuento de leucocitos de 16.030 por mm^3^ con neutrofilia, hemoglobina de 15 g/dl y creatinina de 0,77 mg/dl; el electrocardiograma, el ionograma, los tiempos de coagulación y las pruebas de función hepática, fueron normales. Se le tomó una tomografía de cráneo con resultados también normales.

Al segundo día de la ingestión, continuaba requiriendo soporte respiratorio y con el agente vasopresor norepinefrina, y presentaba aumento de reactantes de fase aguda, hipernatremia (152 mmol/L) e hipocalcemia (0,75 mmol/L), con empeoramiento de los índices de oxigenación, por lo que se hizo una tomografía de tórax que registró hallazgos sugestivos de aspiración bronquial en el lóbulo inferior derecho, por lo que se inició la administración de ampicilina-sulbactam.

Al cuarto día de evolución, cursó con fiebre de 38,8 °C, hipertensión arterial, mioclonías y creatina-fosfocinasa (CPK) elevada (6.520 U/L); 24 horas después, la fiebre persistía, por lo que se modificó el tratamiento antibiótico. Además, seguía presentando alteración del grado de conciencia, a pesar de la suspensión de la sedación y la analgesia; se le hizo un electroencefalograma que evidenció cambios por encefalopatía sin actividad epileptiforme.

**Figura 1:**
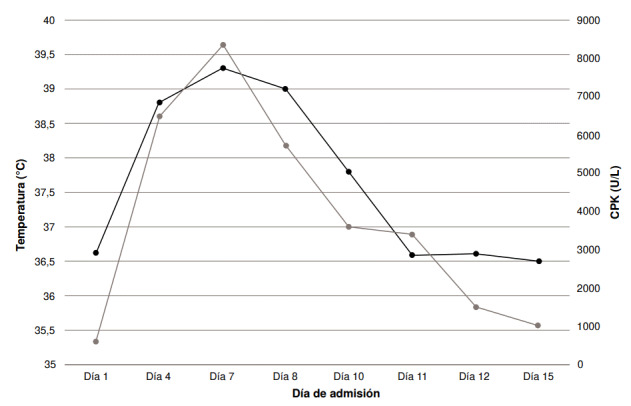
Relación de la evolución de los valores de la creatina fosfocinasa (creatine phosphokinase, CPK) (línea gris) y la temperatura en grados Celsius (línea negra)

A los siete días, el paciente persistía con disfunción autonómica, fiebre hasta de 39,0 °C, rigidez en «tubo de plomo» y flictenas en ambos lados de la cara y los brazos; aunque no estaba sedado, seguía letárgico, lo que se asoció con la leucocitosis (10.110 por mm^3^) y la elevación persistente de la CPK (figura 1), a pesar del uso de cristaloides y manitol. Por ello, se decidió iniciar la administración de bromocriptina en dosis de 5 mg por vía oral cada 8 horas, progresivamente aumentada hasta los 30 mg por día; 48 horas después hubo mejoría del estado neurológico, sin nuevos picos febriles, por lo que se hizo la extubación con éxito.

Cuando se interrogó de nuevo a la familia, se encontró que el paciente había ingerido un pesticida. Se solicitó la cuantificación de las colinestarasas séricas, las cuales se encontraron en menos de 1.500 U/L (valor de referencia: 7.000 a 19.000 U/L), y de la colinesterasa eritrocitaria, la cual se reportó en 157 UPH/h (valor de referencia: 91 a 164 UPH/h). No se administraron oximas, plasma fresco congelado o emulsiones lipídicas. Después de la recuperación del estado neurológico, el paciente confirmó la ingestión intencional del insecticida organofosforado clorpirifós (O,O-dietil-0- 3,5,6-tricloro-2-piridinil-fosforotioato) de uso agrícola. El paciente fue dado de alta sin secuelas 16 días después de la ingestión del pesticida.

### Consideraciones éticas

Este estudio se apegó a lo señalado por la Declaración de Helsinki y lo dispuesto en la Ley 8430 de 1993 de Colombia, por la cual se establecen las normas científicas, técnicas y administrativas para la investigación en salud. Se obtuvo aval del comité de ética hospitalario, así como consentimiento informado del paciente, y se protegió la confidencialidad de la información.

## Discusión

El síndrome neuroléptico maligno es una condición rara y potencialmente letal que ha sido asociada principalmente con el uso de antipsicóticos de primera y segunda generación, así como al de antieméticos, antiparkinsonianos, metiltirosina, tetrabenazina, citalopram, anfetaminas, carbonato de litio, fenitoína y ácido valproico, entre otros ^3^^,^^4^. El reconocimiento temprano es complejo y se han descrito múltiples criterios diagnósticos ^5^.

Todavía no se entiende completamente el mecanismo fisiopatológico del síndrome neuroléptico maligno; algunas teorías vinculan su aparición con el bloqueo del receptor de dopamina-2 en el sistema nervioso central, especialmente en la vía nigroestriada, lo que explica la aparición de la rigidez y el temblor ^3^^,^^6^. También, se ha postulado la hipótesis de la hiperactividad simpático-suprarrenal inducida como un efecto tóxico directo periférico en el músculo esquelético, lo que deteriora la función mitocondrial y el influjo de calcio intracelular, e induce rigidez e hipertermia secundarias ^4^.

Los organofosforados son los insecticidas responsables del mayor número de intoxicaciones por plaguicidas en Colombia y otros países del mundo; tienen un potencial efecto neurotóxico, descrito desde 1987, que explica los grados variables de compromiso neurológico, los cuales van desde la agitación hasta las convulsiones y el coma. En estudios experimentales, se ha demostrado su efecto en la liberación exagerada de dopamina del cuerpo estriado, por un mecanismo dependiente de vesículas, pero independiente de sus transportadores, y la acción de mediadores como glutamato y óxido nítrico ^6^. Nuestra hipótesis es que la aparición de este síndrome en la intoxicación por clorpirifós podría deberse a su toxicidad a causa de los radicales libres que llevan a la destrucción de células neuronales dopaminérgicas y, por ende, a su disminución en el sistema nigroestriado ^7^^-^^9^.

En una revisión de la neurotoxicidad por organofosforados, se evidenció que, después de una crisis colinérgica aguda, solo el 0,5 % de los pacientes desarrolla manifestaciones neurotóxicas en forma de síndromes extrapiramidales, como rigidez, crisis oculógiras, temblores, bradicinesia y distonía, con un inicio de los síntomas a los cuatro días de la intoxicación y un rango de duración de 25 días a 2 meses; el 71 % de estos pacientes se recupera incluso sin necesidad de fármacos antiparkinsonianos ^10^.

En el presente caso, se reporta la cronología de la enfermedad en un hombre de edad media que ingresó al servicio de urgencias con alteración del grado de conciencia después de ingerir intencionalmente un tóxico y tras una semana de la intoxicación cumplía plenamente con los tres criterios clínicos mayores propuestos en 1985 por Levenson para el síndrome neuroléptico maligno ^7^: fiebre, rigidez y elevación de la CPK, y con los menores: taquicardia, alteración sensorial, diaforesis, presión arterial lábil o elevada, diaforesis, hipernatremia y leucocitosis.

Se estableció, asimismo, un porcentaje de actividad enzimática del 21 % de las colinesterasas séricas en relación con el límite inferior normal propuesto por nuestro laboratorio. Posteriormente, aunque en forma tardía, se confirmó la ingestión de un inhibidor de colinesterasas. Se revisó el registro de los medicamentos administrados al paciente y no se encontraron agentes comúnmente relacionados con el síndrome neuroléptico maligno. Sus manifestaciones clínicas no se asociaron a condiciones como neuroinfección, tétanos, estatus epiléptico no convulsivo, trastornos endocrinos, hipertermia maligna, síndrome serotoninérgico o catatonia maligna, ni con otros tóxicos ^4^.

Los reportes de casos similares son pocos. Ochi, *et al.*, publicaron la historia de un hombre de 60 años con antecedentes de esquizofrenia quien, tras la ingestión de metidatión (ditiofosfato de S-2,3-dihidro-5-metoxi-2-oxo- 1,3,4-tiadiazol-3-ilmetilo y O,O-dimetilo), presentó alteración del grado de conciencia, broncorrea, depresión respiratoria y convulsiones, y fue tratado con lavado gástrico, atropina y pralidoxima. Después de la recuperación inicial, en el día 13 de su estancia hospitalaria, presentó fiebre, rigidez “en tubo de plomo” y elevación de la CPK, pero mejoró al cabo de tres días de tratamiento con dantrolene, su evolución clínica fue similar a la del presente caso.

## Conclusión

Aunque en la literatura científica hay reportes de síntomas extrapiramidales posteriores a una intoxicación, el presente es el segundo caso publicado en español o inglés de un síndrome neuroléptico maligno secundario a la intoxicación por organofosforados, posible desde el punto de vista biológico por la relación del clorpirifós con la desregulación de la neurotransmisión dopaminérgica, por lo que se debe considerar en el espectro de sus complicaciones clínicas para garantizar su diagnóstico y tratamiento tempranos.
